# Generation of small molecule-binding RNA arrays and their application to fluorogen-binding RNA aptamers

**DOI:** 10.1016/j.ymeth.2019.04.021

**Published:** 2019-09-01

**Authors:** Charlotte A. Henderson, Callum A. Rail, Louise E. Butt, Helen A. Vincent, Anastasia J. Callaghan

**Affiliations:** School of Biological Sciences and Institute of Biological and Biomedical Sciences, University of Portsmouth, Portsmouth PO1 2DY, United Kingdom

**Keywords:** RNA array, RNA-small molecule interactions, Fluorogen-binding RNA aptamer, Malachite green aptamer, Spinach, Fluorescent biosensors, MG_SA_, malachite green-binding RNA aptamer tagged with a streptavidin-binding RNA aptamer, miRNAs, microRNAs, PBS, phosphate-buffered saline, PBST, phosphate-buffered saline containing 0.05% (v/v) Tween 20, SAM, S-adenosyl methionine, SELEX, systematic evolution of ligands by exponential enrichment, Spinach_SA_, Spinach aptamer tagged with a streptavidin-binding RNA aptamer, Spinach/SAM_SA_, Spinach/SAM RNA biosensor tagged with a streptavidin-binding RNA aptamer, sRNAs, small RNAs, TB, thermodynamically balanced, TBIO, thermodynamically balanced inside-out, TPP, thymine pyrophosphate, UTR, untranslated region

## Abstract

The discovery and engineering of more and more functions of RNA has highlighted the utility of RNA-targeting small molecules. Recently, several fluorogen-binding RNA aptamers have been developed that have been applied to live cell imaging of RNA and metabolites as RNA tags or biosensors, respectively. Although the design and application of these fluorogen-binding RNA aptamer-based devices is straightforward in theory, in practice, careful optimisation is required. For this reason, high throughput *in vitro* screening techniques, capable of quantifying fluorogen-RNA aptamer interactions, would be beneficial. We recently developed a method for generating functional-RNA arrays and demonstrated that they could be used to detect fluorogen-RNA aptamer interactions. Specifically, we were able to visualise the interaction between malachite green and the malachite green-binding aptamer. Here we expand this study to demonstrate that functional-RNA arrays can be used to quantify fluorogen-aptamer interactions. As proof-of-concept, we provide detailed protocols for the production of malachite green-binding RNA aptamer and DFHBI-binding Spinach RNA aptamer arrays. Furthermore, we discuss the potential utility of the technology to fluorogen-binding RNA aptamers, including application as a molecular biosensor platform. We anticipate that functional-RNA array technology will be beneficial for a wide variety of biological disciplines.

## Highlights

• Techniques for quantitative *in vitro* analysis of fluorogen-RNA aptamers are needed.
• We have recently developed a novel method for the production of functional-RNA arrays.
• A protocol for the production of fluorogen-binding RNA aptamer arrays is presented.
• We assess the malachite green-binding aptamer and Spinach aptamer using RNA arrays.
• We demonstrate application of RNA arrays to biosensors using a Spinach/SAM RNA array.

## 1 Introduction

The repertoire of RNA function is far greater than simply that of genetic information transducer originally suggested by the DNA-to-RNA-to-protein central dogma [1]. Recently reviewed by Breaker and Joyce, there are now many established roles for non-coding RNAs [2]. For example, RNA can serve as an adapter molecule (tRNA [3], [4]); it can base-pair with other RNAs to regulate gene expression through a wide variety of mechanisms (bacterial small RNAs (sRNAs), reviewed in [5]; eukaryotic microRNAs (miRNAs), reviewed in [6]); it can have enzymatic properties (natural ribozymes [7], [8], including the ribosome [9], [10]; engineered ribozymes [11]); it can selectively bind molecules/macromolecules with high affinity (aptamers [12], [13]); and it can combine ligand-binding with enzymatic activity (aptazymes, reviewed in [14]) and gene regulation (natural riboswitches, reviewed in [15]; engineered riboswitches, reviewed in [16], [17]).

As more and more functions of RNA are discovered, or engineered, the prevalence of RNA-small molecule interactions, and their potential utility, is apparent. The earliest examples of RNA-targeting small molecules were the antibacterial translation inhibitors that have been used both therapeutically and as functional probes (reviewed in [18], [19]). The growing number of potential RNA targets linked to disease has resulted in considerable interest in pharmaceutical targeting of RNA by small molecules, in a manner analogous to drug-discovery programs that target proteins (reviewed in [20], [21], [22]). In addition, synthetic biologists are interested in regulatory RNA-based molecular sensors. Although natural sensors exist, for example riboswitches, which control gene expression through the adoption of mutually exclusive structures in the presence or absence of ligand (reviewed in [15]) and allosteric ribozymes, which cleave RNA upon binding of a small molecule (reviewed in [14]), the majority of devices to-date have been engineered. Methods for the *in vitro* evolution of RNAs that bind a specific small molecule, aptamers, were described in 1990 [12], [13]. Such aptamers can be subsequently incorporated into ribozymes, to produce aptazymes, which allows small molecule-dependent control of chemical reactions (reviewed in [14]) or into the 5′ untranslated region (UTR) of genes, to generate synthetic riboswitches for gene expression control (reviewed in [16], [17]). More recently, fluorogen-binding “light-up” RNA aptamers have been engineered that result in fluorescence that can be used to monitor RNA in live cells or be applied as molecular biosensors (reviewed in [23], [24], [25], [26]).

Several fluorogen-binding RNA aptamer systems have been generated using systematic evolution of ligands by exponential enrichment (SELEX; [12], [13]) to identify the RNA aptamers (e.g. malachite green-binding aptamer [27], DFHBI-binding Spinach [28], TO1-binding Mango [29]). Second-generation RNA aptamers have also been engineered using rational design and limited screening (Spinach2 [30]) or by combining the SELEX approach with high-throughput functional screening (Broccoli [31]). Likewise, when engineering biosensor devices based on fluorogen-binding RNA aptamers, a functional screening step is required. These biosensors combine a “sensing” non-fluorogenic/non-fluorescent small molecule-binding RNA aptamer with an “output” fluorogen-binding RNA aptamer that results in a fluorescent readout in response to a non-fluorescent signal. Although it was originally hoped that the sensing and output modules would function as discrete units that could be combined, and recombined, at will, in reality careful optimisation of transducer regions is required for device functionality (reviewed in [26], [32]). Given the resource costs involved in optimising and screening *in vivo*, high-throughput *in vitro* screening techniques that can be applied to fluorogen-binding RNA aptamers would be beneficial.

We recently developed a method for generating functional-RNA arrays that could be used as a platform technology for studying RNA-based interactions [33]. Briefly, DNA *in vitro* transcription templates, each encoding a functional-RNA of interest coupled to an immobilisation RNA aptamer are immobilised onto a DNA capture surface in an array format. A DNA *in vitro* transcription template array – *in vitro* transcription reagent solution – RNA capture surface “sandwich” is then assembled. As *in vitro* transcription proceeds, enzymatically synthesised RNAs are captured on the RNA capture surface, via their immobilisation aptamer tag, to produce a functional-RNA array. Competing RNA array technologies are also available. These utilise photolithography coupled with either chemical synthesis of DNA *in vitro* transcription templates followed by enzymatic synthesis of the RNA [34], [35] or chemical synthesis of the RNA [36] to generate an RNA array. However, the reliance of these approaches on chemical synthesis of nucleic acids, either of the DNA *in vitro* transcription template or the RNA itself, has limited these methods to the production of RNA arrays of short RNA oligomers [34], [35], [36]. In contrast, our method allows for the synthesis, folding and immobilisation of both short RNA oligomers and functional-RNAs up to several kb in length. We have successfully produced functional-RNA arrays of non-coding RNAs (e.g. small molecule-binding RNA aptamers, sRNAs and 5’ UTRs of bacterial mRNAs) [33] and mRNAs (e.g. mCherry) [37]. This, in turn, allows for the investigation of physiologically relevant interactions [33], [37].

Using functional-RNA arrays produced by our method, we have demonstrated the detection of cognate sRNA-mRNA pairing for several bacterial sRNAs and also the direct fluorescence-based detection of malachite green by the malachite green-binding RNA aptamer [33]. The latter was particularly promising given the current interest in RNA-targeting small molecules, including fluorogen-binding RNA aptamers. Here we discuss the capabilities of our functional-RNA arrays in investigating fluorogen-binding to RNA aptamers, with particular focus on quantification. We also outline the possible applications of functional-RNA arrays in the design and utility of fluorogen-binding RNA aptamer systems and biosensors based upon them.

## 2 Methods and approaches

### 2.1 Overview

**Fig. 1:**
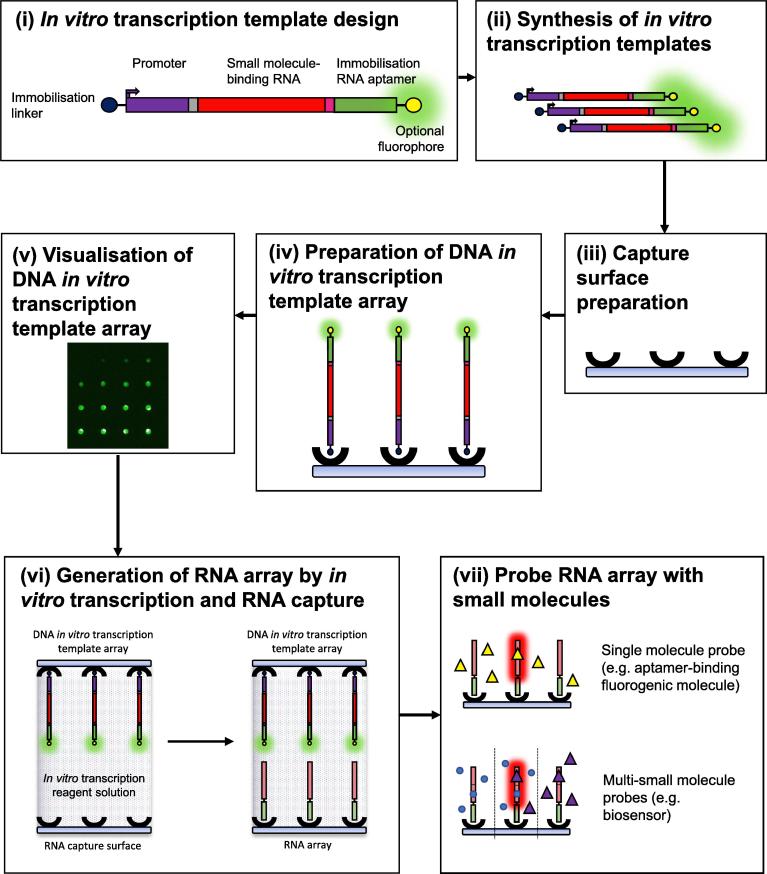
**Flow chart outlining the method for the generation and application of small molecule-binding RNA arrays.** (i) Double-stranded DNA *in vitro* transcription templates, encoding a small molecule-binding RNA of interest coupled to an immobilisation RNA aptamer, and tagged at their 5′ end with an immobilisation linker and at their 3′ end with an optional fluorophore, are designed. (ii) *In vitro* transcription templates are synthesised. (iii) DNA and RNA capture surfaces are prepared. (iv) A DNA *in vitro* transcription template array is generated by immobilising the DNA *in vitro* transcription templates onto the DNA capture surface via the 5′ immobilisation linker in an array format. (v) If a fluorophore tag has been included at the 3′ end of the DNA *in vitro* transcription template, the DNA *in vitro* transcription template array is visualised. (vi) A “sandwich” consisting of a DNA *in vitro* transcription template array — *in vitro* transcription reagent solution — RNA capture surface is assembled (left hand side). As *in vitro* transcription proceeds, RNAs are captured on the RNA capture surface via the immobilisation RNA aptamer, generating an RNA array (right hand side). (vii) The RNA array is probed with either a fluorogenic small molecule (for fluorogen-binding RNA aptamer systems with applications in molecular engineering and fluorogen-binding RNA aptamer tags) (upper image) or both a non-fluorogenic and a fluorogenic small molecule (for applications involving fluorogen-binding RNA-aptamer based biosensors) (lower image).

A flow-chart outlining each of the steps involved in our method for the generation and application of functional-RNA arrays to small molecule-binding RNAs is presented in Fig. 1. Each step will be described in detail in methods and approaches 2.2, 2.3, 2.4, 2.5, 2.8 below and so will only be described briefly here. Initially, DNA *in vitro* transcription templates encoding a small molecule-binding RNA of interest coupled to an immobilisation RNA aptamer, tagged with a 5′ immobilisation linker and an optional 3′ fluorophore, are designed and synthesised (Fig. 1(i) and (ii)). These are immobilised onto a DNA capture surface in array format to generate a DNA *in vitro* transcription template array (Fig. 1(iii) to (v)). A DNA *in vitro* transcription template array — *in vitro* transcription reagent solution — RNA capture surface “sandwich” is assembled (Fig. 1(vi) left hand side). As *in vitro* transcription proceeds, RNAs are captured on the RNA capture surface via the immobilisation RNA aptamer, generating a functional-RNA array (Fig. 1(vi) right hand side). Dependent on the application, the functional-RNA array is probed with either a fluorogenic small molecule (for fluorogen-binding RNA aptamer systems with applications in molecular engineering and fluorogen-binding RNA aptamer tags) (Fig. 1(vii) upper image) or with both a non-fluorogenic and a fluorogenic small molecule (with applications to fluorogen-binding RNA-aptamer based biosensors) (Fig. 1(vii) lower image).

### 2.2 DNA *in vitro* transcription template design

**Fig. 2:**
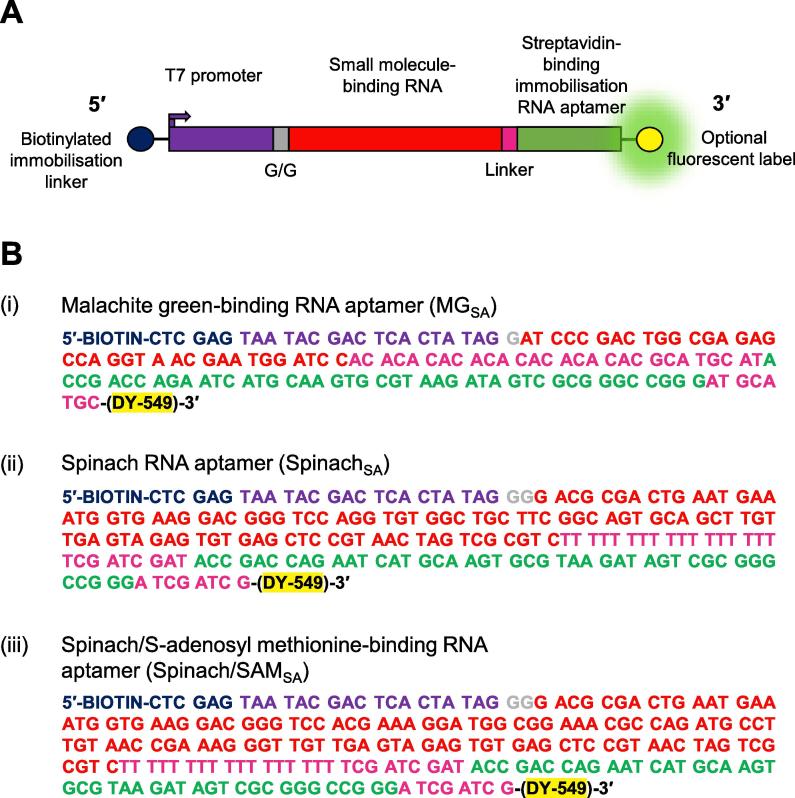
**DNA *in vitro* transcription template design.** (A) A schematic of the overall *in vitro* transcription template design. From 5′ to 3′ this includes: a biotinylated immobilisation linker (dark blue); a minimal T7 promoter (purple); up to two G nucleotides (grey; see text for more information); sequence encoding the small molecule-binding RNA of interest (red); sequence encoding an unstructured linker (pink); sequence encoding the streptavidin-binding immobilisation RNA aptamer (green); an optional fluorescent label (yellow). (B) DNA sequence details for (i) malachite green-binding, (ii) Spinach and (iii) Spinach-based S-adenosyl methionine (SAM)-binding RNA aptamer *in vitro* transcription templates, coloured according to A.

The design of the *in vitro* transcription template is critical for the successful generation of a small molecule-binding functional-RNA array. Each template is based on a common overall design (Fig. 2A). This consists of a 5′ biotinylated immobilisation linker (5′–biotin–ctc gag–3′), a minimal T7 promoter (5′–taa tac gac tca cta tag–3′), sequence encoding a small molecule-binding RNA of interest, an unstructured linker, a sequence encoding the streptavidin-binding immobilisation RNA aptamer (5′–acc gac cag aat cat gca agt gcg taa gat agt cgc ggg ccg gg–3′) and an optional 3′ fluorescent tag.

For optimal performance, the DNA *in vitro* transcription template array should consist of templates that are immobilised on the capture surface via an interaction with their 5′ end. 5′ end site-specific immobilisation serves the dual purpose of facilitating oriented immobilisation and also maximises the access of *in vitro* transcription reaction components to the template. A short linker with a reactive amino functional group at its 5′ end can be used to immobilise the DNA *in vitro* transcription templates on an NHS-activated surface by amine coupling [38], [39]. However, it is challenging to identify conditions that selectively immobilise the DNA via this linker [38], [39]. Therefore, we typically achieve selective 5′ immobilisation using a streptavidin-coated surface as the DNA capture surface and including a short 5′ biotinylated linker, of essentially arbitrary sequence (we use 5′–biotin–ctc gag–3′), at the 5′ end of the DNA *in vitro* transcription template.

A promoter sequence is required for *in vitro* RNA synthesis by RNA polymerase. For proof-of-concept studies, we have elected to use the T7 *in vitro* transcription system due to its yield, versatility and commercial availability. However, dependent on the application, it may be advisable to use alternative transcription systems. We will discuss some situations when this may by desirable in the results and applications section of this manuscript. We envisage that other *in vitro* transcription systems could be employed provided that the *in vitro* transcription template is modified to include the system-compatible promoter/promoter elements.

The small molecule-binding RNA sequences are user-defined and will depend on the experimental purpose. For example, a minimal binding motif, such as a hairpin, may be sufficient to detect small molecule binding but a full-length functional-RNA, such as an mRNA, may be needed to investigate transcription performance. To-date, we have demonstrated that RNA arrays consisting of a range of non-coding RNAs (including small molecule-binding RNA aptamers, sRNAs and the 5′ UTRs of bacterial mRNAs) [33] or functional mRNAs (mCherry mRNA) [37] can be produced. Small molecule-binding RNA sequences may need to be modified for optimal transcription. For example, T7 RNA polymerase requires a G in the +1 position (included in the minimal promoter sequence) and is enhanced by a G in both the +2 and +3 positions [40]. We routinely modify the RNA sequences to include Gs in positions +1, +2 and +3 unless there are indications that modification is likely to affect the structure of the RNA. The potential effects of modification on RNA structure are assessed using RNA structure prediction programs such as Mfold [41] or RNAfold from the ViennaRNA Package [42]. The DNA sequences used in this study, to demonstrate the applications to small molecule-binding RNAs, are presented in Fig. 2B.

Once synthesised, the small molecule-binding RNA must bind to an RNA capture surface to generate the RNA array. We decided to use immobilisation RNA aptamers encoded 3′ to the small molecule-binding RNA to facilitate the tethering of the small molecule-binding RNA to a capture surface coated with the immobilisation RNA aptamer’s cognate ligand. The use of a structured RNA aptamer in the 3′ location serves as an internal control for RNA folding and ensures that only fully-transcribed small molecule-binding RNAs are immobilised. Critical considerations when selecting an immobilisation RNA aptamer include the affinity for the cognate ligand; the availability of the cognate ligand; and the availability of surface-immobilisation strategies for the cognate ligand. We have demonstrated proof-of-concept with the tobramycin-binding RNA aptamer [33] and the streptavidin-binding RNA aptamer [33], [37]. Both the streptavidin-binding and tobramycin-binding RNA aptamers have nanomolar affinity for their cognate ligand and have been used successfully as affinity purification tags [43], [44]. Furthermore, both streptavidin and tobramycin are commercially available and can be immobilised via standard amine coupling chemistry. However, the streptavidin-binding RNA aptamer consistently performed better than the tobramycin-binding RNA aptamer [33] and so we typically use a streptavidin-binding RNA aptamer and a streptavidin-coated RNA capture surface.

Since function of the small molecule-binding RNA and the streptavidin-binding RNA aptamer will depend on them adopting their correct structures, an unstructured linker is included between the small molecule-binding RNA and the streptavidin-binding RNA aptamer to promote the independent folding of the two RNAs. A variety of linker sequences have been used successfully and these are presented in Table 1. We recommend that RNA structure prediction programs such as Mfold and RNAfold [41], [42] are used when designing *in vitro* transcription templates to evaluate the potential effects of the linker sequence on small molecule-binding RNA and streptavidin-binding RNA aptamer folding.

**Table 1:** Suggested small molecule-binding RNA–immobilisation RNA aptamer linker sequences.

Aptamer	DNA sequence	Ref.
Tobramycin	5′–aca cac aca cac aca cac ac–3′	[33]
	5′–ata tcc ccc ccc ccc ccc cc–3′	[33]
	5′–ttt ttt ttt tcc ccc ccc cc–3′	[33]
	5′–aaa aaa aaa aaa aaa aaa a–3′	[33]

Streptavidin	5′–aca cac aca cac aca cac acg cat gca t–(aptamer)–atg cat gc–3′	[33], [37], *This study*
	5′–ttt ttt ttt ttt ttt ttt ttt gtg tg–(aptamer)–cac aca–3′	[33]
	5′–ttt ttt ttt ttt ttt ttt tag ag–(aptamer)–ctc ta–3′	[33]
	5′–ttt ttt ttt ttt ttt ttt gtg tg–(aptamer)–cac ac–3′	[33]
	5′–aat aat aat aat aat aat aat atg cat gc–(aptamer)–gca tgc at–3′	[33]
	5′–ttt ttt ttt ttt ttt ttt atg cat gc–(aptamer)–gca tgc at–3′	[33]
	5′–ttt ttt ttt ttt ttt ttt cga tcg at–(aptamer)–atc gat cg–3′	*This study*

Finally, the optional 3′ fluorescent tag allows for visualisation and quantification of the DNA *in vitro* transcription templates on the array. We use a 5′ fluorescently labelled reverse primer complementary to the 3′ end of the sequence encoding the streptavidin-binding RNA aptamer to incorporate the fluorescent tag during template synthesis. A variety of fluorophores can be incorporated in this manner and choice of fluorophore will be dependent on the detection capabilities of the microarray slide scanner to be used. We typically use Dy-549.

### 2.3 Synthesis of DNA *in vitro* transcription templates

The 5′ biotinylated, or 5′ biotinylated and 3′ Dy-549-labelled, double-stranded DNA *in vitro* transcription templates must conform to the overall design principles discussed in methods and approaches Section 2.2. Dependent on the length of the small molecule-binding RNA, they can be generated in a number of ways, including annealing of complementary oligodeoxyribonucleotides [45], PCR/gene synthesis [46] or cloning and restriction digestion of a plasmid-based construct [47]. Detailing each of these methods, which should be familiar to a molecular biologist and have been reported in the context of the production of RNA arrays previously [33], [37], is beyond the scope of this manuscript. Instead, we will focus on the gene synthesis protocol used to generate the DNA *in vitro* transcription templates used in this manuscript.

#### 2.3.1 Materials

DNA oligonucleotide primers (Invitrogen; see Table 2 for sequences).

**Table 2:** Primer sequences used in the preparation of DNA *in vitro* transcription templates.

*In vitro* transcription template	Primer	Sequence
MG_SA_	TBIO gene synthesis S2	5′–taa tac gac tca cta tag gat ccc gac tgg cga gag cca ggt aac gaa tgg atc–3′
	TBIO gene synthesis S1	5′–gag cca ggt aac gaa tgg atc cac aca cac aca cac aca cac gca tgc ata ccg a–3′
	TBIO gene synthesis AS1	5′–cgg ccc gcg act atc tta cgc act tgc atg att ctg gtc ggt atg cat gcg tgt g–3′
	TBIO gene synthesis AS2	5′–gca tgc atc ccg gcc cgc gac tat ctt–3′

Spinach_SA_	TB gene synthesis S1	5′–ctc gag taa tac gac tca cta tag ggg acg cga ctg aat gaa atg gtg aag gac ggg tcc a–3′
	TB gene synthesis AS1	5′–cgg agc tca cac tct act caa caa gct gca ctg ccg aag cag cca cac ctg gac ccg tcc ttc acc att–3′
	TB gene synthesis S2	5′–tga gta gag tgt gag ctc cgt aac tag tcg cgt ctt ttt ttt ttt ttt ttt tcg atc gat acc gac ca–3′
	TB gene synthesis AS2	5′–cga tcg atc ccg gcc cgc gac tat ctt acg cac ttg cat gat tct ggt cgg tat cga tcg aaa aaa–3′

Spinach/SAM_SA_	TB gene synthesis S1	5′–ctc gag taa tac gac tca cta tag ggg acg cga ctg aat gaa atg gtg aag gac ggg tcc acg aaa gga–3′
	TB gene synthesis AS1	5′–gct cac act cta ctc aac aac cct ttc ggt tac aag gca tct ggc gtt tcc gcc atc ctt tcg tgg acc cgt cg–3′
	TB gene synthesis S2	5′–gtt gtt gag tag agt gtg agc tcc gta act agt cgc gtc ttt ttt ttt ttt ttt ttt cga tcg ata ccg acc ag–3′
	TB gene synthesis AS2	5′–cga tcg atc ccg gcc cgc gac tat ctt acg cac ttg cat gat tct ggt cgg tat cga tcg aaa a–3′

MG_SA_, Spinach_SA_, Spinach/SAM_SA_	Biotinylation Forward	5′–biotin–ctc gag taa tac gac tca cta tag–3′

MG_SA_	Fluorescent label Reverse	5′–Dy-549–gca tgc atc ccg gcc cgc gac tat ctt–3′

Spinach_SA_, Spinach/SAM_SA_	Fluorescent label Reverse	5′–Dy-549–cga tcg atc ccg gcc cgc gac tat ctt acg cac ttg cat gat tct ggt cgg tat cga tcg aaa a–3′

KOD Hot Start DNA Polymerase (Sigma; 71086-3).

dNTPs (Thermo Scientific; R0193).

Macherey-Nagel NucleoSpin Gel and PCR Clean-up Kit (Macherey-Nagel; 740609).

#### 2.3.2 Equipment

GeneAmp PCR System 9700 thermocycler (Applied Biosystems).

#### 2.3.3 Protocol

Non-labelled PCR templates are initially generated by gene synthesis [48]. Overlapping primers are designed using DNAWorksv3.2.4 (U. S. National Institutes of Health, Bethesda, Maryland, USA; https://hpcwebapps.cit.nih.gov/dnaworks/) which, by default, designs primers based on thermodynamically balanced (TB) gene synthesis. Variable parameters are set to a T_m_ of 62 °C and oligo lengths between 60 and 80 nucleotides. If TB gene synthesis is unsuccessful, primers can be redesigned in DNAWorks using TBIO (thermodynamically balanced inside-out) mode. The primers that were used for gene synthesis in this manuscript are presented in Table 2. Gene synthesis is then performed using KOD Hot Start DNA polymerase in a thermocycler (95 °C for 2 min; 25x 95 °C for 20 s, 53 °C for 30 s, 70 °C for 30 s; 70 °C for 5 min; 4 °C hold). The products are analysed by agarose gel electrophoresis. The 5′ biotin and 3′ Dy-549 labels, required for surface immobilisation and detection/quantification, respectively, are added by amplifying correctly sized products with a common 5′ biotinylated forward and template-specific 5′ Dy-549-labelled reverse primer by PCR (see Table 2 for primer sequences; KOD Hot Start DNA polymerase; 95 °C for 2 min; 25x 95 °C for 20 s, 53 °C for 30 s, 70 °C for 30 s; 70 °C for 5 min; 4 °C hold). Products are purified using a NucleoSpin Gel and PCR clean-up kit and analysed by agarose gel electrophoresis. Care should be taken to keep the Dy-549-labelling reactions and labelled products in the dark.

### 2.4 Preparation of streptavidin-coated capture surfaces

Both the production of the DNA *in vitro* transcription template array and the small molecule-binding RNA array require a streptavidin-coated capture surface. It is important to be aware that the use of streptavidin-coated capture surfaces for both the DNA *in vitro* transcription templates and the small molecule-binding RNAs could result in the *in vitro* transcribed RNA binding to the DNA capture surface, rather than to the RNA capture surface, as intended. We minimise any potential problems associated with this by controlling the density of streptavidin on the capture surfaces. Therefore, although streptavidin-coated microarray slides are commercially available, we prepare them using NHS-activated microarray slides, commercially available purified streptavidin and standard amine coupling chemistry. This approach allows us to prepare an RNA capture surface with a higher density of streptavidin than the DNA capture surface. Alternative solutions include using orthogonal capture-systems for the DNA and RNA, or the DNA capture surface could be blocked with biotin following DNA capture.

#### 2.4.1 Materials

NEXTERION Slide H NHS-activated microarray slides (Schott; 1070936)

LifterSlips (Thermo Scientific; 22X25I-2-4816 and 24X60I-2-4733).

Streptavidin (Sigma; S0677-1MG).

Phosphate-buffered saline (PBS) (Fisher Scientific; BP399).

Tween 20 (Fisher Scientific; BP337).

Ethanolamine (Sigma; E9508).

#### 2.4.2 Equipment

Humidified chamber comprising a Labnet Mini incubator (Labnet International, I5110A), containing a glass bowl with 25 ml water.

Denley Spiramix rolling platform (Thermo Scientific).

#### 2.4.3 Protocol

**Fig. 3:**
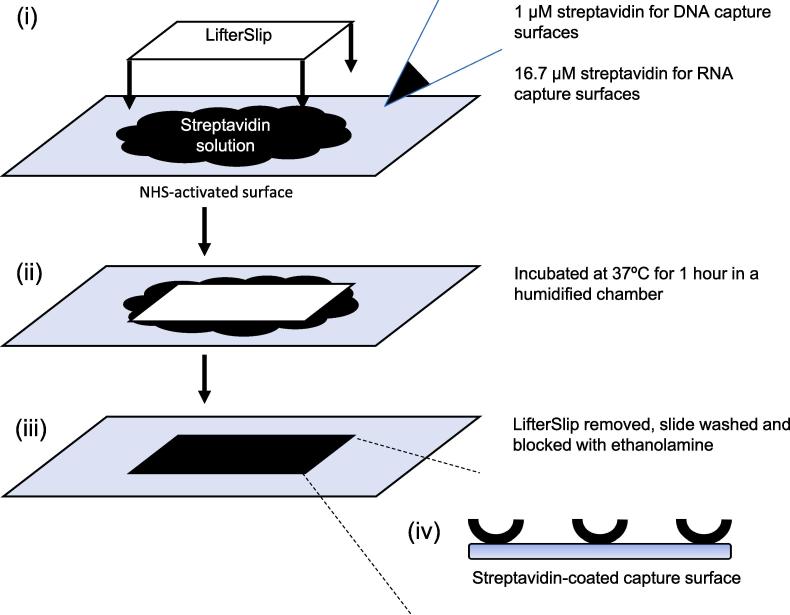
**A schematic of streptavidin-coated DNA and RNA capture surface preparation.** (i) Streptavidin (1 μM for DNA and 16.7 μM for RNA capture surfaces, respectively) in phosphate-buffered saline (PBS), pH 7.4 (black) is pipetted onto an NHS-activated surface (pale blue) and covered with a LifterSlip (white). (ii) The assembly is incubated at 37 °C for 1 hr in a humidified environment. (iii) The resultant streptavidin-coated capture surface following LifterSlip removal, washing and surface-blocking. (iv) Zoom-in of the resultant streptavidin-coated capture surface with individual streptavidin molecules represented by black semicircular arcs.

90 µl of 1 µM streptavidin (DNA capture surface) or 16.7 µM streptavidin (RNA capture surface) in PBS, pH 7.4, is pipetted onto a NHS-activated microarray slide (Fig. 3). This is covered with a LifterSlip and incubated at 37 °C for 1 h in a humidified environment (we use a mini incubator containing a bowl of water). The LifterSlip is removed, the resultant streptavidin-coated capture surface is placed in a 50 ml Falcon tube and washed according to the following wash cycle: (i) 45 ml PBS containing 0.05% (v/v) Tween 20 (PBST) for 5 min, with rolling, at room temperature (ii) 45 ml PBS for 5 min, with rolling, at room temperature and (iii) 45 ml H_2_O for 5 min, with rolling, at room temperature. The streptavidin-coated capture surface is then incubated in 45 ml 50 mM ethanolamine-HCl pH 8.5 for 30 min, with rolling, at room temperature, to block any unreacted NHS functional groups. A second wash cycle is performed: (i) 45 ml PBST for 5 min, with rolling, at room temperature (ii) 45 ml PBS for 5 min, with rolling, at room temperature and (iii) 45 ml H_2_O for 5 min, with rolling, at room temperature. The streptavidin-coated capture surface is dried by centrifugation at 500 × g for 5 min at room temperature. The streptavidin-coated capture surfaces can be stored at −20 °C for at least two weeks, until required.

### 2.5 Generation of DNA *in vitro* transcription template array

Template arrays of DNA *in vitro* transcription templates are prepared by immobilising 5′ biotinylated *in vitro* transcription templates onto streptavidin-coated DNA capture surfaces in an array format. The biotinylated templates can be spotted onto the streptavidin-coated DNA capture surface manually with a pipette (low- to medium-throughput, between 10 and 0.2 µl spots) or with an automated arrayer (high-throughput, nl spots). In our experience, similar results are obtained from both manual and automated spotting [33]. This is because the sensitivity of the technique depends on the density of the DNA *in vitro* transcription template within a spot rather than on the absolute amount of DNA *in vitro* transcription template present. Reducing the spotting volume, by manually pipetting a smaller volume or using an automated arrayer, also reduces the spot size which offsets any effect on DNA density. The primary advantage of automated spotting is not increased sensitivity, rather, it is the smaller spot size that enables increased spot density and increased throughput. Here we focus on high-spot density arrays that are produced using an automated arrayer. However, the principles described are equally applicable to low- to medium-spot density arrays produced by manual spotting. In the first instance, it is advisable to spot each DNA *in vitro* transcription template at a range of concentrations because variability in transcription efficiency and probe sensitivity can affect the limits of detection. We typically start by spotting DNA *in vitro* transcription template prepared at concentrations ranging from low nM to low μM.

#### 2.5.1 Materials

5′ biotinylated or 5′ biotinylated and 3′ Dy-549-labelled DNA *in vitro* transcription templates (prepared according to methods and approaches Section 2.3).

Streptavidin-coated DNA capture surface (prepared according to methods and approaches Section 2.4).

PBS (Fisher Scientific; BP399).

Tween 20 (Fisher Scientific; BP337).

#### 2.5.2 Equipment

Qarray 2 arrayer (Genetix).

Denley Spiramix rolling platform (Thermo Scientific).

#### 2.5.3 Protocol

**Fig. 4:**
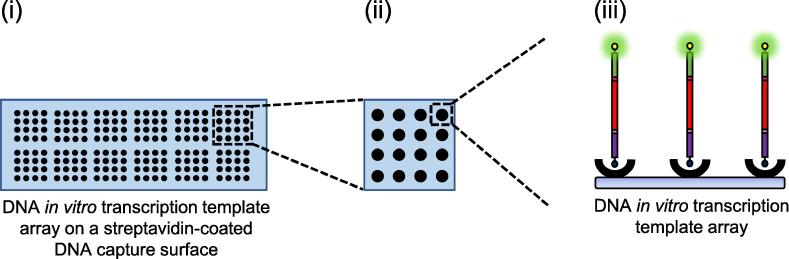
**A schematic illustration of the DNA *in vitro* transcription template array**. (i) The entire DNA *in vitro* transcription template array with *in vitro* transcription template spots shown as black circles. (ii) An enlarged single 4 × 4 field of the DNA *in vitro* transcription template array. (iii) An enlarged single spot of the DNA *in vitro* transcription template array showing streptavidin-surface-capture of individual DNA *in vitro* transcription templates.

An automated arrayer is used to spot a concentration range of 5′ biotinylated, or 5′ biotinylated and 3′ fluorescently labelled *in vitro* DNA template, in PBS, pH 7.4, onto a streptavidin-coated DNA capture surface (Fig. 4). This is incubated in the arrayer for 30 min at 50% relative humidity and at room temperature. This step should be performed under conditions to preserve the stability of the fluorophore. Please note that the Qarray 2 arrayer that we use has a tinted cover for this purpose. Subsequent wash steps are performed in foil-covered Falcon tubes. The resultant DNA *in vitro* transcription template array is briefly dipped in PBST and then washed with: (i) 45 ml PBST for 5 min, with rolling, at room temperature (ii) 45 ml PBS for 5 min, with rolling, at room temperature and (iii) 45 ml H_2_O for 5 min, with rolling, at room temperature. It is dried by centrifugation at 500 × g for 5 min at room temperature. The DNA *in vitro* transcription template array can be stored at −20 °C for at least two weeks, until required.

### 2.6 Visualisation and quantification of DNA *in vitro* transcription template arrays

We have access to a microarray scanner system that includes both the microarray slide scanner and integrated image analysis software. However, densitometry can also be performed using stand-alone image analysis software such as ImageJ (Rasband WS, ImageJ, U. S. National Institutes of Health, Bethesda, Maryland, USA, http://imagej.nih.gov/ij/, 1997–2016).

#### 2.6.1 Equipment

GenePix 4300A microarray scanner with integrated GenePix Pro 7 image analysis software (Molecular Devices).

#### 2.6.2 Protocol

Dy-549 fluorescently-labelled DNA *in vitro* transcription template arrays are visualised with a microarray scanner using an excitation wavelength of 532 nm and a Standard Green filter. The fluorescence intensity of each spot on the array is quantified using the integrated image analysis software on the microarray scanner.

### 2.7 Generation of small molecule-binding RNA arrays

**Fig. 5:**
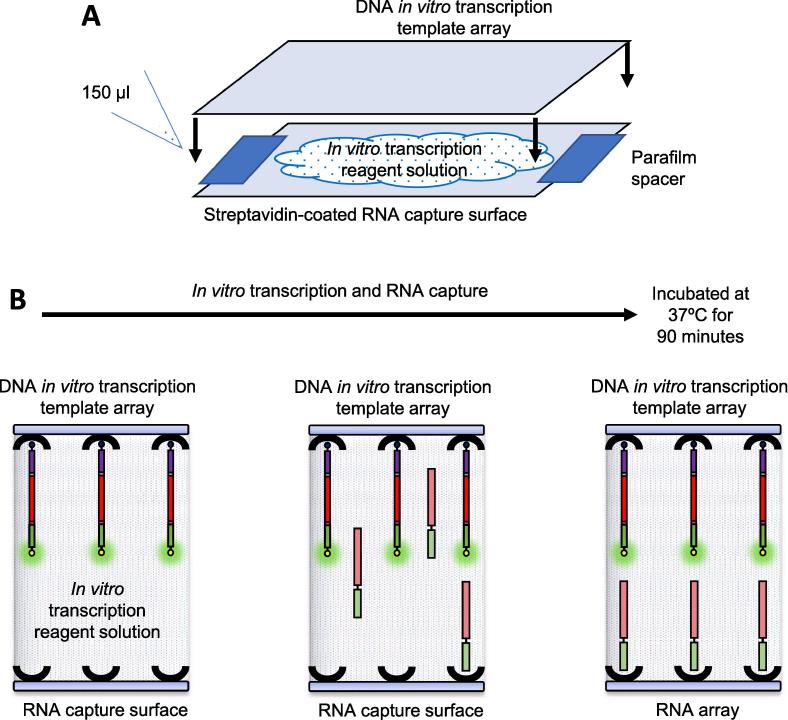
**A schematic illustration of the sandwich assembly method used to produce small molecule-binding RNA arrays.** (A) The DNA *in vitro* transcription template array – *in vitro* transcription reagent solution – streptavidin-coated RNA capture surface sandwich assembly. The streptavidin-coated RNA capture surface is placed streptavidin-coated surface side up. Parafilm spacers are positioned at each end of the surface. 150 μl *in vitro* transcription reagent solution is pipetted onto the streptavidin-coated capture surface. The DNA *in vitro* transcription template array, array side down, is lowered onto the streptavidin-coated RNA capture surface. (B) Progress of *in vitro* transcription and RNA capture during incubation at 37 °C for 90 min. On the left is the DNA *in vitro* transcription template array – *in vitro* transcription reagent solution – streptavidin-coated RNA capture surface sandwich assembly at 0 min; no RNA has been *in vitro* transcribed or captured. The middle panel represents the DNA *in vitro* transcription template array – *in vitro* transcription reagent solution – streptavidin-coated RNA capture surface sandwich assembly at an intermediate time between 0 and 90 min; *in vitro* transcription is occurring and RNA is being captured on the RNA capture surface. Finally, on the right is the DNA *in vitro* transcription template array – *in vitro* transcription reagent solution – streptavidin-coated RNA capture surface sandwich assembly at 90 min; *in vitro* transcription and RNA capture are complete.

Small molecule-binding RNA arrays are produced using a “sandwich” assembly of a DNA *in vitro* transcription template array – *in vitro* transcription reagent solution – streptavidin-coated RNA capture surface (Fig. 5). Parafilm spacers are used to separate the two surfaces. These help to reduce the surface tension of the sandwich assembly and this aids disassembly of the sandwich once *in vitro* transcription is complete. *In vitro* transcription conditions can be varied but a good starting condition is incubation at 37 °C for 90 min.

#### 2.7.1 Materials

DNA *in vitro* transcription template array (prepared according to methods and approaches Section 2.5).

Streptavidin-coated RNA capture surface (prepared according to methods and approaches Section 2.4).

Parafilm (Bemis Flexible Packaging).

MEGAscript T7 Transcription Kit (Invitrogen; AMB1334-5).

PBS (Fisher Scientific; BP399).

Tween 20 (Fisher Scientific; BP337).

#### 2.7.2 Equipment

Humidified chamber comprising a Labnet Mini incubator (Labnet International, I5110A), containing a glass bowl with 25 ml water.

#### 2.7.3 Protocol

The DNA *in vitro* transcription template array – *in vitro* transcription reagent solution – streptavidin-coated RNA capture surface “sandwich” is assembled as shown in Fig. 5A. A streptavidin-coated RNA capture surface slide is positioned streptavidin-coated surface side up with parafilm spacers at each of the surface’s short ends. 150 µl *in vitro* transcription reagent solution (1X MEGAscript T7 Transcription Reaction Buffer, 1X MEGAscript T7 Transcription Enzyme Mix, 0.4 mM each of ATP, CTP, GTP and UTP) is pipetted onto the RNA capture surface. A DNA *in vitro* transcription template array is positioned on top of the RNA capture surface, array side down. This assembly is incubated in a humidified environment at 37 °C for 90 min (Fig. 5B). The sandwich is disassembled and the newly generated small molecule-binding RNA array is placed in a 50 ml Falcon tube and washed with: (i) 45 ml PBST for 5 min, with rolling, at room temperature (ii) 45 ml PBS for 5 min, with rolling, at room temperature and (iii) 45 ml H_2_O for 5 min, with rolling, at room temperature. It is then dried by centrifugation at 500 × g for 5 min at room temperature.

### 2.8 Probing the small molecule-binding RNA array with small molecules

In order to visualise RNA-small molecule interactions using RNA arrays there must be a mechanism for detecting the RNA-small molecule interaction. The simplest option is direct visualisation of the interaction using fluorescence. As discussed in the introduction, several fluorogen-binding RNA aptamer systems have been developed for use as RNA tags or molecular biosensors (reviewed in [23], [24], [25], [26]. Fluorogen-binding RNA arrays can be probed either *in situ* during RNA array production, by supplementing the *in vitro* transcription reagent solution with the fluorogen, or post RNA array production, by incubating the RNA array with a probing solution containing the fluorogen. In this manuscript we utilise the malachite green-binding aptamer [27], the DFHBI-binding Spinach aptamer [28] and the Spinach-based S-adenosyl methionine (SAM) biosensor [49] to demonstrate the capabilities of small molecule-binding RNA arrays. The choice of probing protocol and optimal probing conditions vary depending on the specific fluorogen-RNA interaction due to factors such as binding affinity, binding kinetics and photophysical properties of the fluorogen-RNA aptamer complex. Therefore, the specific probing conditions required for these three fluorogen-binding RNA aptamer systems will be discussed in detail in the results and applications section and only general probing protocols will be presented here.

#### 2.8.1 Probing small molecule-binding RNA arrays *in situ* during RNA array production

##### 2.8.1.1 Materials

DNA *in vitro* transcription template array (prepared according to methods and approaches Section 2.5).

Streptavidin-coated RNA capture surface (prepared according to methods and approaches Section 2.4).

Parafilm (Bemis Flexible Packaging).

MEGAscript T7 Transcription Kit (Invitrogen; AMB1334-5).

Probe (see results and applications section for details).

PBS (Fisher Scientific; BP399).

Tween 20 (Fisher Scientific; BP337).

##### 2.8.1.2 Equipment

Humidified chamber comprising a Labnet Mini incubator (Labnet International, I5110A), containing a glass bowl with 25 ml water.

GenePix 4300A microarray scanner with integrated GenePix Pro 7 image analysis software (Molecular Devices).

##### 2.8.1.3 Protocol

The DNA *in vitro* transcription template array – *in vitro* transcription reagent solution – streptavidin-coated RNA capture surface “sandwich” is assembled as shown in Fig. 5A. A streptavidin-coated RNA capture surface is positioned streptavidin-coated surface side up with parafilm spacers at each of the surface’s short ends. 150 µl *in vitro* transcription reagent solution (1X MEGAscript T7 Transcription Reaction Buffer, 1X MEGAscript T7 Transcription Enzyme Mix, 0.4 mM each of ATP, CTP, GTP and UTP) supplemented with the appropriate small molecule probe(s) (see results and applications section for details) is pipetted onto the RNA capture surface. A DNA *in vitro* transcription template array is positioned on top of the RNA capture surface, array side down. This assembly is incubated in a humidified environment at 37 °C for 90 min. The sandwich is disassembled and the newly generated, probed small molecule-binding RNA array is placed in a 50 ml Falcon tube and washed with: (i) 45 ml PBST for 5 min, with rolling, at room temperature (ii) 45 ml PBS for 5 min, with rolling, at room temperature and (iii) 45 ml H_2_O for 5 min, with rolling, at room temperature. It is then dried by centrifugation at 500 × g for 5 min at room temperature. The probed small molecule-binding RNA array is visualised using the appropriate excitation wavelength and emission filter for the fluorogen-binding RNA system (see results and applications section for details) using a microarray slide scanner. The fluorescence intensity of each spot on the array is quantified using the integrated image analysis software on the microarray scanner.

#### 2.8.2 Probing small molecule-binding RNA arrays post RNA array production

##### 2.8.2.1 Materials

LifterSlips of 24 mm × 60 mm (Thermo Scientific; 24X60I-2-4733).

Probe (see results and applications section for details).

Probe buffer (see results and applications section for details).

##### 2.8.2.2 Equipment

Humidified chamber comprising a Labnet Mini incubator (Labnet International, I5110A), containing a glass bowl with 25 ml water.

GenePix 4300A microarray scanner with integrated GenePix Pro 7 image analysis software (Molecular Devices).

##### 2.8.2.3 Protocol

**Fig. 6:**
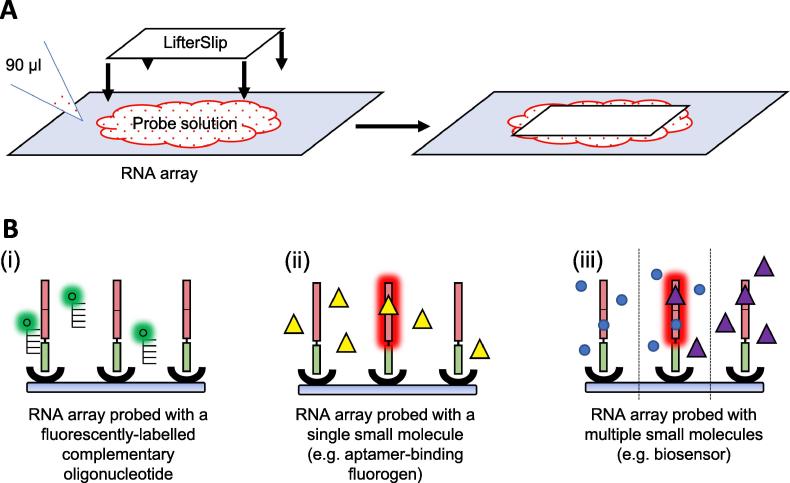
**A schematic of probing of RNA arrays post RNA array production.** (A) The probing assembly. 90 μl of probe in probe buffer is pipetted onto the small molecule-binding RNA array and covered with a LifterSlip. (B) (i) The RNA array probed using a fluorescently labelled oligonucleotide complementary to the linker region between the small molecule-binding RNA and the streptavidin-binding immobilisation RNA aptamer. (ii) The RNA array probed using a single aptamer-binding fluorogenic small molecule (yellow triangles). (iii) The RNA array probed using a combination of an aptamer-binding non-fluorogenic small molecule (blue circles) and an aptamer-binding fluorogenic small molecule (purple triangles) (e.g. for biosensors).

90 µl of small molecule probe in probe buffer (see results and applications section for details) is pipetted onto the small molecule-binding RNA array and covered with a LifterSlip (Fig. 6). This is incubated in the dark, in a humidified environment at room temperature. Generally, the LifterSlip is removed, and the probed small molecule-binding RNA array is placed in a 50 ml Falcon tube and washed before being dried by centrifugation at 500 × g for 5 min at room temperature (see results and application section for details). The probed small molecule-binding RNA array is visualised using the appropriate excitation wavelength and emission filter for the fluorogen-binding RNA system (see results and application section for details) using a microarray slide scanner. The fluorescence intensity of each spot on the array is quantified using the integrated image analysis software on the microarray scanner.

## 3 Results and applications

### 3.1 RNA-targeting fluorogenic small molecules

#### 3.1.1 Malachite green and the malachite green-binding aptamer

**Fig. 7:**
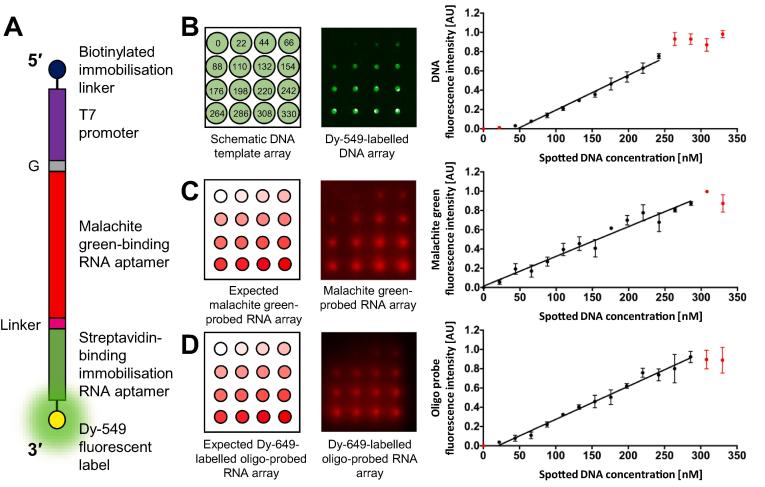
**A malachite green-binding RNA aptamer (MG_SA_) RNA array probed post RNA array production with malachite green.** (A) A schematic of the MG_SA_ DNA *in vitro* transcription template. (B) (Left panel) A schematic of a 4 × 4 field of a MG_SA_ DNA *in vitro* transcription template array to be spotted. The DNA concentration to be spotted at each position is indicated, in nM. (Centre panel) A 4 × 4 field of a Dy-549-labelled MG_SA_ DNA *in vitro* transcription template array spotted according to the schematic shown in the left panel using an automated arrayer fitted with a 175 μm pinhead, allowing for a spot separation of 1125 μm. (Right panel) A plot of DNA fluorescence intensity against spotted DNA concentration. (C) (Left panel) A schematic of the mirror image of a 4 × 4 field of the expected MG_SA_ RNA array probed post RNA array production with malachite green. (Centre panel) A mirror image of a 4 × 4 field of the MG_SA_ RNA array probed post RNA array production with 40 μM malachite green. (Right panel) A plot of malachite green fluorescence intensity against spotted DNA concentration. (D) (Left panel) A schematic of the mirror image of a 4 × 4 field of the expected MG_SA_ RNA array probed post RNA array production with a Dy-649-labelled complementary oligonucleotide probe. (Centre panel) A mirror image of a 4 × 4 field of the MG_SA_ RNA array probed post RNA array production with 50 nM Dy-649-labelled oligonucleotide probe (5′–Dy-649–gtg tgt gtg tgt gtg tgt gt–3′). (Right panel) A plot of oligonucleotide probe fluorescence intensity against spotted DNA concentration. For each of the plots, the data are the mean from at least three separate fields of the respective array and error bars represent the standard deviation. Data were fitted to linear equations and data outside the linear range are indicated in red.

We recently demonstrated that the malachite green aptamer–malachite green interaction could be detected on an RNA array [33]. This was a qualitative study, providing a simple yes/no output. Here we show that this interaction is quantifiable (Fig. 7). Following the protocols outlined in the methods and approaches section, DNA *in vitro* transcription template arrays of Dy-549 fluorescently-labelled or non-labelled templates, encoding the malachite green-binding aptamer (MG_SA_), were prepared (Fig. 2B(i) and 7A). The templates were prepared and spotted at a range of concentrations between 22 and 330 nM using an automated arrayer (Fig. 7B). For the Dy-549-labelled MG_SA_ DNA *in vitro* transcription template array, fluorescence intensity was linear for DNA template concentrations between 44 and 242 nM (Fig. 7B).

Non-labeled MG_SA_ DNA *in vitro* transcription template arrays were *in vitro* transcribed to generate MG_SA_ RNA arrays. Probing of the MG_SA_ RNA array *in situ* during RNA array production by supplementing the *in vitro* transcription reagent solution with 40 μM malachite green was unsuccessful. There are several possible explanations for why this didn’t work. For example, malachite green may affect the *in vitro* transcription reaction or, the *in vitro* transcription reaction buffer may be suboptimal for malachite green binding. However, since MG_SA_ RNA arrays can be probed post RNA array production, as discussed below, we did not perform further troubleshooting. For post RNA array production probing, we typically use between 20 and 40 µM malachite green in 40 mM Tris-HCl (pH 7.8), 20 mM NaCl, 6 mM MgCl_2_ and probe for 2 to 16 h at room temperature. The RNA array is then placed in a foil-covered 50 ml Falcon tube and is washed with: (i) 45 ml H_2_O for 1 min, with rolling, at room temperature; (ii) 45 ml of H_2_O for 10 s, with rolling, at room temperature. This is followed by a final dip in 45 ml H_2_O at room temperature before drying by centrifugation at 500 × g for 5 min at room temperature. Probing of a MG_SA_ RNA array with 40 µM malachite green (Sigma; 38800) for 2 h at room temperature post RNA array production resulted in detectable fluorescence from the malachite green-binding RNA aptamer–malachite green complex using an excitation wavelength of 635 nm and a Standard Red filter (Fig. 7C). Quantification of the fluorescence intensity of the malachite-green probed RNA array revealed a positive correlation between the concentration of DNA *in vitro* transcription template spotted on the array and the malachite green fluorescence emitted. This correlation was linear over a DNA concentration range from 22 to 286 nM (Fig. 7C).

The fluorescence emitted following probing with malachite green appears to provide a readout of the amount of functional RNA present on the MG_SA_ RNA array. In order to confirm this, a MG_SA_ RNA array, prepared as described above, was probed with a fluorescently-labelled oligonucleotide complementary to the linker region between the malachite green-binding and streptavidin-binding RNA aptamers (Fig. 7D). A protocol for probing RNA arrays with complementary oligonucleotides has been reported previously [37]. Following this protocol, the MG_SA_ RNA array was probed post RNA array production with 50 nM linker probe (5′–Dy-649–gtg tgt gtg tgt gtg tgt gt–3′; Invitrogen) in 2X SSC, 0.1% (w/v) SDS for 30 min at room temperature. The probed MG_SA_ RNA array was placed in a foil-covered 50 ml Falcon tube and was washed with: (i) 45 ml PBST for 5 min, with rolling, at room temperature; (ii) 45 ml H_2_O for 30 s, with rolling, at room temperature. This was followed by a final dip in 45 ml H_2_O at room temperature before drying by centrifugation at 500 × g for 5 min at room temperature. Detectable fluorescence was observed using an excitation wavelength of 635 nm and a Standard Red filter (Fig. 7D). The fluorescence intensity was linear relative to DNA *in vitro* transcription template concentrations between 22 and 286 nM (Fig. 7D). These data suggest that the malachite green probe enables RNA quantification over the same DNA *in vitro* transcription template concentration range as the fluorescently-labelled oligonucleotide probe.

#### 3.1.2 DFHBI and the Spinach aptamer

**Fig. 8:**
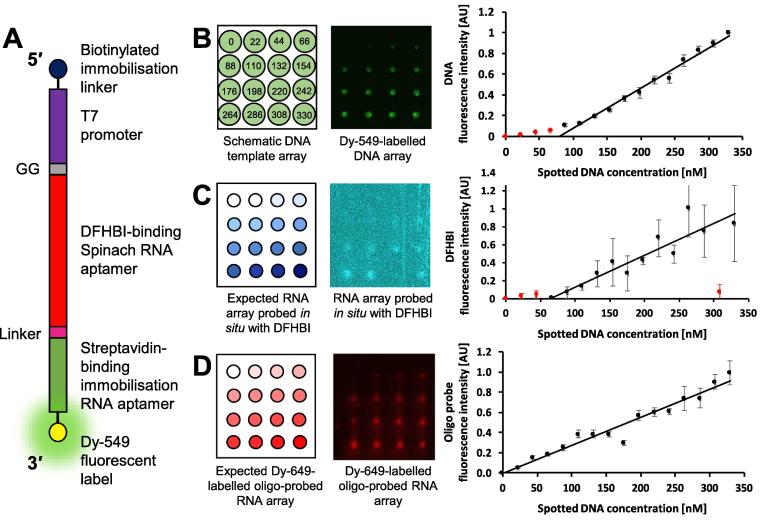
**A Spinach aptamer (Spinach_SA_) RNA array probed *in situ* with DFHBI.** (A) A schematic of the Spinach_SA_ DNA *in vitro* transcription template. (B) (Left panel) A schematic of a 4 × 4 field of a Spinach_SA_ DNA *in vitro* transcription template array to be spotted. The DNA concentration to be spotted at each position is indicated, in nM. (Centre panel) A 4 × 4 field of a Dy-549-labelled Spinach_SA_ DNA *in vitro* transcription template array spotted according to the schematic shown in the left panel using an automated arrayer fitted with a 175 μm pinhead, allowing for a spot separation of 1125 μm. (Right panel) A plot of DNA fluorescence intensity against spotted DNA concentration. (C) (Left panel) A schematic of the mirror image of a 4 × 4 field of the expected Spinach_SA_ RNA array probed *in situ* during RNA array production with DFHBI. (Centre panel) A mirror image of a 4 × 4 field of the Spinach_SA_ RNA array probed *in situ* during RNA array production with 152 μM DFHBI. The array has been false-coloured cyan to aid visualisation. (Right panel) A plot of DFHBI fluorescence intensity against spotted DNA concentration. (D) (Left panel) A schematic of the mirror image of a 4 × 4 field of the expected Spinach_SA_ RNA array probed post RNA array production with a Dy-649-labelled complementary oligonucleotide probe. (Centre panel) A mirror image of a 4 × 4 field of the Spinach_SA_ RNA array probed post RNA array production with 50 nM Dy-649-labelled oligonucleotide probe (5′–Dy-649–aaa aaa aaa aaa aaa aaa aa–3′). (Right panel) A plot of oligonucleotide probe fluorescence intensity against spotted DNA concentration. For each of the plots, the data are the mean from at least three separate fields of the respective array and error bars represent the standard deviation. Data were fitted to linear equations and data outside the linear range are indicated in red.

In order to demonstrate that our approach is applicable to other RNA-targeting fluorogenic small molecules we performed a similar series of experiments based on the DFHBI-binding Spinach aptamer. Following the protocols outlined in the methods and approaches section, DNA *in vitro* transcription template arrays of Dy-549 fluorescently-labelled or non-labelled templates, encoding the Spinach aptamer (Spinach_SA_), were prepared (Fig. 2B(ii) and 8A). The templates were spotted at a range of concentrations between 22 and 330 nM using an automated arrayer (Fig. 8B). For the Dy-549-labelled Spinach_SA_ DNA *in vitro* transcription template array, fluorescence intensity was linear for DNA template concentrations between 88 and 330 nM (Fig. 8B).

Non-labeled Spinach_SA_ DNA *in vitro* transcription template arrays were *in vitro* transcribed to generate Spinach_SA_ RNA arrays. A Spinach_SA_ RNA array was probed *in situ* during RNA array production by supplementing the *in vitro* transcription reagent solution with 152 μM DFHBI (Merck; SML1627-5MG) and visualising using an excitation wavelength of 488 nm and a Standard Blue filter (Fig. 8C). A positive linear correlation was observed between the concentration of DNA *in vitro* transcription template spotted on the array and the DFHBI–Spinach_SA_ fluorescence over a DNA concentration range from 66 to 330 nM.

To confirm that the DFHBI–Spinach fluorescence is representative of the amount of Spinach_SA_ on the RNA array, a Spinach_SA_ RNA array was also probed with a fluorescently-labelled oligonucleotide complementary to the linker region between the Spinach and streptavidin-binding RNA aptamers (Fig. 8D). The Spinach_SA_ RNA array was probed post RNA array production with 50 nM linker probe (5′–Dy-649–aaa aaa aaa aaa aaa aaa–3′; Invitrogen) and visualised as described for the MG_SA_ aptamer in Section 3.1.1 above. The fluorescence intensity was linear relative to DNA *in vitro* transcription template concentrations between 22 and 330 nM (Fig. 8D). These data suggest that the DFHBI probe enables RNA quantification over a slightly narrower DNA *in vitro* transcription template concentration range than the fluorescently-labelled oligonucleotide probe.

#### 3.1.3 Applications

##### 3.1.3.1 Development and optimisation of fluorogen-binding RNA aptamers

Each of the fluorogen-binding RNA aptamers developed to-date were engineered using SELEX. They were then subjected to various degrees of optimisation of the RNA aptamer sequence and/or the fluorogen. The detection and quantification capabilities of RNA arrays with respect to fluorogen-binding RNA aptamers implies that RNA arrays would be a useful tool for the development of both existing and novel fluorogen-binding RNA aptamers. For example, we envisage that RNA arrays of aptamer variants could be produced and tested against a particular fluorogen, providing information about optimal fluorogen-aptamer pairing and orthogonality.

##### 3.1.3.2 Fluorogen-binding RNA aptamer-based transcription assay

The utility of fluorogen-binding RNA aptamers as RNA tags that can be used to monitor RNA levels *in vivo* has been demonstrated (reviewed in [23], [24], [25]). Similarly, quantifiable fluorogen-binding RNA aptamers could be used as RNA tags to assay RNA transcription *in vitro*. This might be particularly useful for the protein expression and synthetic biology fields where understanding, and being able to control, promoter activity as well as the impact of transcription factors and/or transcription inhibitors, is critical for optimal system performance. Thus, RNA arrays comprised of a series of different promoters, for example, could be a useful analytical tool in this instance. It is important to note that, for this application, it may be necessary to employ alternative *in vitro* transcription systems to the T7 system described here to ensure compatibility with the transcription components to be tested.

### 3.2 RNA-targeting non-fluorogenic small molecules

Investigating RNA-targeting non-fluorogenic small molecules using RNA arrays is much more challenging than investigating RNA-targeting fluorogenic small molecules since it requires an indirect detection method or biosensor. One possible approach is to couple a non-fluorogenic small molecule-binding RNA aptamer to a fluorogenic small molecule-binding RNA aptamer so that in the presence of both the non-fluorogenic small molecule and the fluorogen, fluorescence is emitted (reviewed in [26], [32], [50]). In this manner, the DFHBI-binding Spinach RNA aptamer has been combined with a number of non-fluorogenic small molecule-binding RNA aptamers including SAM, adenine, guanine, ADP, GTP, thymine pyrophosphate (TPP), cyclic-di-GMP and cyclic AMP-GMP [49], [51], [52]. As proof-of-concept we decided to use the Spinach/SAM biosensor previously described by Paige et al. [49].

**Fig. 9:**
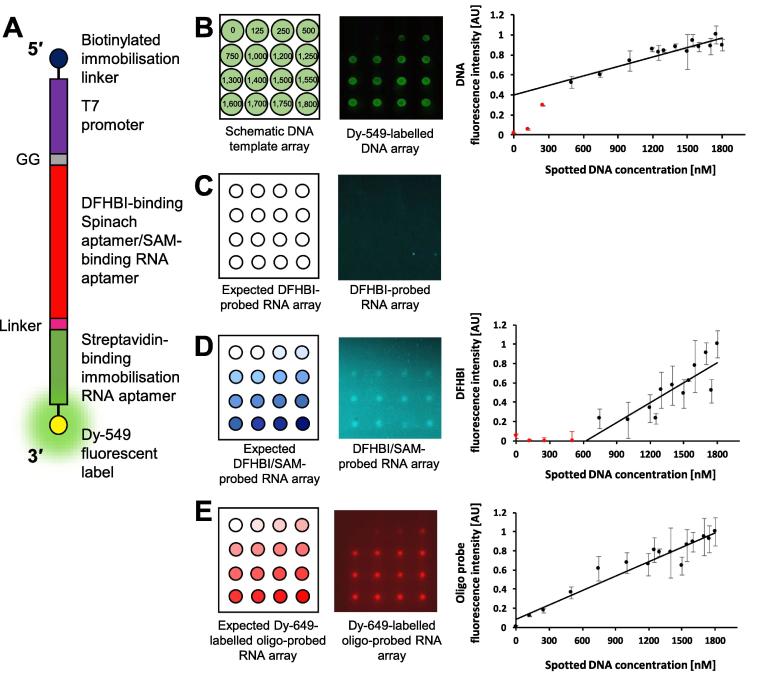
**A Spinach/SAM biosensor (Spinach/SAM_SA_) RNA array probed post RNA array production with DFHBI and SAM.** (A) A schematic of the Spinach/SAM_SA_ DNA *in vitro* transcription template. (B) (Left panel) A schematic of a 4 × 4 field of a Spinach/SAM_SA_ DNA *in vitro* transcription template array to be spotted. The DNA concentration to be spotted at each position is indicated, in nM. (Centre panel) A 4 × 4 field of a Dy-549-labelled Spinach/SAM_SA_ DNA *in vitro* transcription template array spotted according to the schematic shown in the left panel using an automated arrayer fitted with a 175 μm pinhead, allowing for a spot separation of 1125 μm. (Right panel) A plot of DNA fluorescence intensity against spotted DNA concentration. (C) (Left panel) A schematic of the mirror image of a 4 × 4 field of the expected Spinach/SAM_SA_ RNA array probed post RNA array production with DFHBI. (Right panel) A mirror image of a 4 × 4 field of the Spinach/SAM_SA_ RNA array probed post RNA array production with 180 μM DFHBI. The array has been false-coloured cyan to aid visualisation. (D) (Left panel) A schematic of the mirror image of a 4 × 4 field of the expected Spinach/SAM_SA_ RNA array probed post RNA array production with DFHBI and SAM. (Centre panel) A mirror image of a 4 × 4 field of the Spinach/SAM_SA_ RNA array probed post RNA array production with 180 μM DFHBI and 1 mM SAM. The array has been false-coloured cyan to aid visualisation. (Right panel) A plot of DFHBI fluorescence intensity against spotted DNA concentration. (E) (Left panel) A schematic of the mirror image of a 4 × 4 field of the expected Spinach/SAM_SA_ RNA array probed post RNA array production with a Dy-649-labelled complementary oligonucleotide probe. (Centre panel) A mirror image of a 4 × 4 field of the Spinach/SAM_SA_ RNA array probed post RNA array production with 50 nM Dy-649-labelled oligonucleotide probe (5′–Dy-649–aaa aaa aaa aaa aaa aaa aa–3′). (Right panel) A plot of oligonucleotide probe fluorescence intensity against spotted DNA concentration. For each of the plots, the data are the mean from at least three separate fields of the respective array and error bars represent the standard deviation. Data were fitted to linear equations and data outside the linear range are indicated in red.

Following the general protocols outlined in the methods and approaches section, Dy-549 fluorescently-labelled or non-labelled DNA *in vitro* transcription templates encoding the Spinach/SAM biosensor (Spinach/SAM_SA_), were prepared (Fig. 2B(iii) and 9A). The templates were spotted at a range of concentrations between 125 and 1800 nM using an automated arrayer (Fig. 9B). This array was *in vitro* transcribed to generate a Spinach/SAM_SA_ RNA array. Initial attempts at *in situ* probing of the Spinach/SAM_SA_ RNA array were unsuccessful. Therefore, the Spinach/SAM_SA_ RNA array was probed post RNA production with 180 µM DFHBI (Fig. 9C) or with 180 µM DFHBI and 1 mM SAM (Sigma; A7007) (Fig. 9D) in 40 mM HEPES, pH 7.5, 125 mM KCl, 1 mM MgCl_2_ and visualised after 15 min, with the LifterSlip still in place, using an excitation wavelength of 488 nm (50% power) and a Standard Blue filter (Fig. 9C and D). The post probing protocol for Spinach_SA_ arrays required significant modification of both the probing conditions and the visualisation method, relative to probing the MG_SA_ RNA array with malachite green, to account for the known poor stability of the DFHBI-Spinach complex *in vitro* [53]. Since Spinach/SAM_SA_ requires SAM to bind before it can adopt the structure required for DFHBI to bind [49], as expected in the absence of SAM and the presence of DFHBI, no fluorescence was detected (Fig. 9C). In contrast, in the presence of both SAM and DFHBI, fluorescence was clearly detected. However, fluorescence was only detected at DNA *in vitro* template concentrations greater than 750 nM (Fig. 9D). Above this concentration, the fluorescence intensity was linear relative to DNA *in vitro* transcription template concentrations up to the highest concentration tested (1800 nM) (Fig. 9D).

To confirm the RNA levels, the Spinach/SAM_SA_ RNA array was washed with: (i) an initial dip in 40 mM Tris-HCl, pH 7.8, 20 mM NaCl, 6 mM MgCl_2_; (ii) 45 ml 40 mM Tris-HCl, pH 7.8, 20 mM NaCl, 6 mM MgCl_2_ for 1 min, with rolling, at room temperature. This was followed by a final dip in 45 ml H_2_O at room temperature before drying by centrifugation at 500 × g for 5 min at room temperature.

The array was then probed with 50 nM linker probe (5′–Dy-649–aaa aaa aaa aaa aaa aaa–3′; Invitrogen) in 2X SSC, 0.1% (w/v) SDS for 30 min at room temperature. The probed Spinach/SAM_SA_ RNA array was placed in a foil-covered 50 ml Falcon tube and was washed with: (i) an initial dip in 40 mM Tris-HCl, pH 7.8, 20 mM NaCl, 6 mM MgCl_2_; (ii) 45 ml 40 mM Tris-HCl, pH 7.8, 20 mM NaCl, 6 mM MgCl_2_ for 1 min, with rolling, at room temperature. This was followed by a final dip in 45 ml H_2_O at room temperature before drying by centrifugation at 500 × g for 5 min at room temperature. Detectable fluorescence was observed using an excitation wavelength of 635 nm and a Standard Red filter (Fig. 9E) and was linear in a DNA *in vitro* transcription template range from 125 to 1800 nM. In contrast to the data for the MG_SA_ and Spinach_SA_ aptamers, the data for the Spinach/SAM_SA_ aptamer suggest that probing with DFHBI enables RNA quantification over a narrower DNA *in vitro* transcription template concentration range, relative to the fluorescently-labelled oligonucleotide probe. Perhaps, more strikingly, DFHBI-based detection of the Spinach/SAM_SA_ aptamer required significantly higher DNA *in vitro* transcription template concentrations than used previously for the MG_SA_ aptamer and malachite green or the Spinach_SA_ aptamer and DFHBI. This may reflect the additional challenge of requiring two aptamers to fold correctly and two small molecules to bind for DFHBI-based detection. Nevertheless, these data indicate that, with appropriate optimisation of probing and visualisation conditions, RNA arrays would be suitable for Spinach aptamer-based biosensor applications.

#### 3.2.1 Applications

##### 3.2.1.1 Engineering of fluorogen-binding RNA aptamer-based biosensors

A number of Spinach-based non-fluorescent/fluorogenic small molecule biosensors have been engineered. However, their design required optimisation of the connecting region between the Spinach aptamer and the non-fluorescent/fluorogenic small molecule-binding aptamer (reviewed in [26], [32]). RNA arrays have the potential to facilitate this design process for new biosensors since they would allow a series of designs to be tested in parallel. This may be particularly useful for developing biosensors based on optimised versions of Spinach that have been engineered with *in vitro* applications in mind (e.g. iSpinach [54]).

##### 3.2.1.2 Biomarker detection

As more and more fluorogen-binding RNA aptamer-based biosensors are developed, this opens up the possibility of using RNA arrays as a screening platform for biomarkers. Several diseases have known biomarker fingerprints e.g. cancers [55]. Therefore, the assembly of multiple RNA aptamer-based biosensors corresponding to these fingerprints on a single array platform could prove to be a useful non-invasive diagnostic tool for these diseases.

## 4 Remaining challenges

There are clearly hurdles to overcome before RNA arrays can routinely be applied as diagnostic tools. For example, biosensors will be required that can detect a wide range of biomarkers including small molecules and also larger macromolecules such as proteins and nucleic acids. In addition, there is the challenge of stabilising RNA so that it is not degraded in the presence of biological samples. However, combining SELEX approaches with RNA array screening could guide the development of these biosensors such that the final diagnostic platform could be manufactured using available RNA stabilisation chemistries [56]. In reality, we envisage that small molecule-binding RNA arrays will prove to be of the greatest utility in the engineering and optimisation of small molecule-aptamer systems, including fluorogen-binding RNA aptamer-based biosensors. In this capacity, the requirement for DNA *in vitro* transcription templates to be individually designed and synthesised currently limits the throughput capabilities of RNA arrays. However, computer programs could be developed to aid the design of DNA *in vitro* transcription templates and robotics could be employed for the DNA synthesis step. Automation of these, and possibly subsequent steps, could easily allow the high-throughput potential of RNA array technology to be realised.

## 5 Conclusion

Here we have provided a detailed protocol for generating functional-RNA arrays for the purpose of investigating RNA-targeting small molecules. Specifically, we have demonstrated the capabilities of RNA arrays with respect to fluorogen-binding RNA aptamers and fluorogen-binding RNA aptamer-based biosensors. We have also discussed the potential applications of RNA arrays to the design, optimisation and utility of fluorogen-binding RNA aptamers. We anticipate that, in these capacities, RNA arrays will prove to be a valuable tool for a range of disciplines including molecular biology, metabolic engineering, synthetic biology and medicine.
